# Targeting Premature Renal Aging: from Molecular Mechanisms of Cellular Senescence to Senolytic Trials

**DOI:** 10.3389/fphar.2021.630419

**Published:** 2021-04-29

**Authors:** Rossana Franzin, Alessandra Stasi, Elena Ranieri, Giuseppe Stefano Netti, Vincenzo Cantaluppi, Loreto Gesualdo, Giovanni Stallone, Giuseppe Castellano

**Affiliations:** ^1^ Department of Emergency and Organ Transplantation, Nephrology, Dialysis and Transplantation Unit, University of Bari Aldo Moro, Bari, Italy; ^2^ Clinical Pathology, Center of Molecular Medicine, Department of Medical and Surgical Sciences, University of Foggia, Foggia, Italy; ^3^ Nephrology and Kidney Transplantation Unit, Department of Translational Medicine and Center for Autoimmune and Allergic Diseases (CAAD), University of Piemonte Orientale (UPO), Novara, Italy; ^4^ Nephrology, Dialysis and Transplantation Unit, Advanced Research Center on Kidney Aging (A.R.K.A.), Department of Medical and Surgical Sciences, University of Foggia, Italy

**Keywords:** senescence, renal ageing, mitochondrial dysfunction, DNA damage repair, extracellular vesicles, senolytics, rapamycin, metformin

## Abstract

The biological process of renal aging is characterized by progressive structural and functional deterioration of the kidney leading to end-stage renal disease, requiring renal replacement therapy. Since the discovery of pivotal mechanisms of senescence such as cell cycle arrest, apoptosis inhibition, and the development of a senescence-associated secretory phenotype (SASP), efforts in the understanding of how senescent cells participate in renal physiological and pathological aging have grown exponentially. This has been encouraged by both preclinical studies in animal models with senescent cell clearance or genetic depletion as well as due to evidence coming from the clinical oncologic experience. This review considers the molecular mechanism and pathways that trigger premature renal aging from mitochondrial dysfunction, epigenetic modifications to autophagy, DNA damage repair (DDR), and the involvement of extracellular vesicles. We also discuss the different pharmaceutical approaches to selectively target senescent cells (namely, senolytics) or the development of systemic SASP (called senomorphics) in basic models of CKD and clinical trials. Finally, an overview will be provided on the potential opportunities for their use in renal transplantation during *ex vivo* machine perfusion to improve the quality of the graft.

## Introduction

The global population aged 60 years or over is expected to double by 2050, when it is projected to reach nearly 2.1 billion (World Population Aging, Economic and Social Affairs, United Nations). The increased life expectancy is unavoidably accompanied by a growing portion of the population diagnosed with age-associated kidney diseases (Glassock et al., 2017; Lv & Zhang, 2019).

Kidneys from elderly are associated with structural changes as the loss in renal mass, glomerulosclerosis, glomerular basement membrane thickening, tubular atrophy, interstitial fibrosis, and the arteriosclerosis (Sobamowo and Prabhakar, 2017; Sturmlechner et al., 2017). Furthermore, aged kidneys are characterized by functional impairments as reduced glomerular filtration rate (GFR), decrease in urine concentration, plasma flow, and sodium resorption. In healthy aging conditions, despite the gradual but constant drop in GFR (Delanaye et al., 2019) (5–10% per decade after the age of 35 years), renal function can be preserved by compensatory mechanisms as hypertrophy of unaffected nephrons or by vasodilatory prostaglandins that can moderate excessive vasoconstriction (Glassock and Rule, 2012).

However, beyond their functional reserve capacity, aged kidneys exhibit an increased susceptibility to “a second hit” damage as during acute kidney injury (AKI) occurrence, such as after a nephrotoxic drugs treatment (i.e., contrast agents) or during a bacterial induced systemic inflammatory response (i.e., sepsis or other infections) (Sturmlechner et al., 2017; Valentijn et al., 2018). In the last few years, it has become extremely clear that maladaptive repair after an AKI episode can predispose to chronic kidney disease (CKD), and ultimately, depending on genetic, immunological, and environmental factors, to end-stage renal disease (ESRD) (Basile et al., 2016; Fiorentino et al., 2018; Formica et al., 2018). This concept is quite in contrast with the previous theories that considered AKI, within a normal range, as a reversible process.

Additionally, kidneys from elderly have been demonstrated to display a lower regenerative capacity; when transplanted these organs show a severe vulnerability to ischemia/reperfusion, correlated with poorer outcome and higher incidence of graft loss and rejection (Li et al., 2018; Rosner et al., 2018; Pool et al., 2020).

**FIGURE 1:**
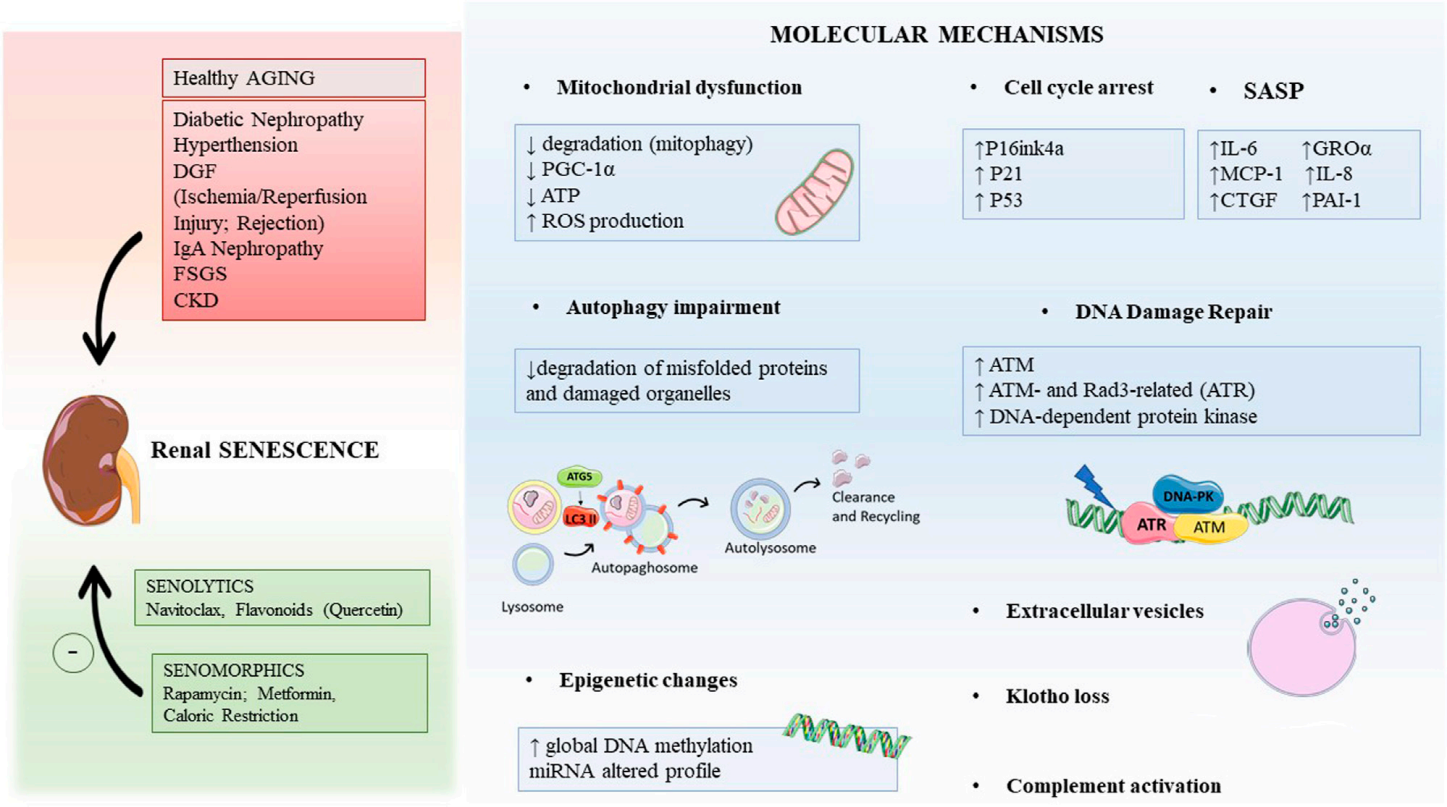
Graphical abstract indicating the main causes leading to renal aging and possible therapeutic intervention strategies (in the left). The key molecular mechanisms inducing renal aging are mitochondrial dysfunction, autophagy, epigenetic changes, DNA damage repair (DDR), extracellular vesicles, and others. Abbreviations: DGF, delay graft function; FSGS, focal segmental glomerulosclerosis; IgAN, IgA nephropathy; CKD, chronic kidney disease; PGC-1α, peroxisome proliferator-activated receptor γ coactivator-1; ROS, oxygen reactive species; GROα, growth-regulated oncogene α; MCP-1, monocyte chemoattractant protein-1; CTGF, connective tissue growth factor; PAI-1, plasminogen activator inhibitor-1; ATM, ataxia telangiectasia mutated.

Besides physiological aging in the elderly, premature pathological aging can be induced by several kidney diseases including diabetic nephropathy, ischemia/reperfusion injury (IRI) and rejection, hypertension and glomerular diseases as membranous nephropathy, IgA nephropathy (IgAN), and focal segmental glomerulosclerosis (FSGS) (Figure 1; Yang and Fogo, 2010; Demaria et al., 2017; Hernandez-Segura et al., 2018; Docherty et al., 2020).

From a molecular perspective, physiological aging of the elderly and premature stress-induced aging share common intracellular pathways and mediators and disentangling the two processes may be difficult (Glassock and Rule, 2012; Sobamowo and Prabhakar, 2017).

The central mechanism underlying renal physiological and pathological aging is characterized by cellular senescence (Hernandez-Segura et al., 2018).

Cellular senescence refers to a complex program that can be initiated by various cellular stresses and is characterized by cycle arrest despite the presence of growth stimuli (Yang and Fogo, 2010; Demaria et al., 2017). In mammals, this process is involved in physiological and beneficial processes such as embryogenesis, tissue regeneration and repair, wound healing, or inhibition of neoplastic transformation. In this “acute setting,” transiently generated senescent cells are then cleared by leucocytes or natural killer cells (Docherty et al., 2019; Docherty et al., 2020). In renal aging-related diseases, senescent cells chronically accumulate in renal parenchyma, leading to tissue deterioration and to an aberrant signaling activation to different types of populations.

The renal senescent cells express several markers such as cell cycle arrest proteins of G1/S and G2/M checkpoint as p16INK4A, p21WAF/CIP1 (encoded by *CDKN1a*), p27KIP1 (encoded by *CDKN1b*), p53 and are missing proliferation markers such as Ki67 (Sturmlechner et al., 2017; Valentijn et al., 2018). For cellular senescence detection, the most commonly used marker is senescence-associated β-galactosidase (SA-β-gal) activity at pH 6.0, which indicates the increased lysosomal activity of senescent cells (Biran et al., 2017). The key characteristics of senescence are the DNA damage response (detected with γ-histone H2AX+ or tumor suppressor p53-binding protein 1 + foci) and senescence-associated heterochromatic foci (SAHF) (Fumagalli et al., 2014). Importantly, senescent cells have a specific secretome-defined SASP that relies on the production of pro-inflammatory cytokines, chemokines, growth factors, and matrix-degrading factor array (i.e., IL-6, IL-1α, IL-1β, GROα, CTGF, plasminogen activator inhibitor 1 (PAI-1), C-C motif chemokine 2 (CCL2, also known as MCP-1)) (Barker et al., 2015; Kooman et al., 2017; Sturmlechner et al., 2017). This secretory phenotype can affect neighboring cells promoting the maintenance of an inflammatory microenvironment and the fibrotic process. However, none of these markers alone can detect senescent cells; therefore, a panel of markers should be used to demonstrate the occurrence of senescence (Docherty et al., 2020).

Based on this pattern on healthy aged kidney biopsies, recent research started to demonstrate that the histology of a normal aged kidney is different from the histology of a diseased kidney, thereby supporting the introduction of an age-adjusted glomerular filtration rate cutoff to define CKD (Melk et al., 2005; Glassock et al., 2020; Rovin, 2020).

Currently, our knowledge coming from the experimental and clinical studies on CKD is still incomplete. Here, we summarize the most recent literature describing the pivotal processes that regulate renal aging in physiological and pathological conditions, underlying their involvement to identify possible new targets for interventional therapies.

## Principal Mechanisms of Renal Aging

Renal aging includes a complex network of signaling from diminished level of nephroprotective factors such as Klotho and bone morphogenic proteins, to the activation of Wnt/β-catenin pathway and oxidative stress, to sirtuins and PPARγ decline (Schmitt and Melk, 2017; Kuro, 2019). These mechanisms with the pro-fibrotic TGFβ1 signaling, the endothelial dysfunction, and the microvascular rarefaction have been extensively reviewed (Docherty et al., 2019). In addition, our group of research recently demonstrated that also complement system, one of the major pillars of innate immune response, is bad for renal aging, as evaluated after ischemia/reperfusion (Castellano et al., 2019; Franzin et al., 2020). Here, we will provide in detail the current updates of additional mechanisms involved in age-related renal diseases such as mitochondrial dysfunction, autophagy, epigenetic modifications, and DNA damage repair (DDR).

### Mitochondrial Dysfunction in Renal Aging

Long recognized for their pivotal role in cellular energy production through cellular respiration and oxidative phosphorylation, mitochondria respond in first line to the high-energy demand of kidneys, which required a higher amount of oxygen and abundance in functional mitochondria to sustain their functions.

Moreover, mitochondria are essential in several cellular mechanisms, including the maintenance of homeostasis in the redox state, the synthesis of several macromolecules, regulation of intracellular calcium, and release of pro- or anti-inflammatory signals and control of intrinsic pathway of apoptosis (Wirthensohn and Guder, 1986; Feucht et al., 2018).

Along nephrons, there is heterogeneity in generating energy; as a matter of fact, proximal tubular cells produce ATP via oxidative phosphorylation, while podocytes, endothelial, and mesangial cells through their glycolytic capacity (Wirthensohn and Guder, 1986). These differences may influence the impact of mitochondrial dysfunction in kidney and in the progression of renal diseases and aging-associated renal failure (Galvan et al., 2017).

In accordance, several studies have demonstrated that both acute and chronic insults lead to alterations in mitochondrial structure, inducing mitochondrial DNA (mtDNA) damage, decreased matrix density, and compromising the outer and inner membrane integrity (Che et al., 2014). The principal features of mitochondrial dysfunction, widely described in several renal diseases and aging, include several changes in mitochondrial formation (biogenesis), remodeling (fusion/fission), degradation (mitophagy), and mitochondrial impaired homeostasis. These alterations lead to a bioenergetic dysfunction, a decrease in ATP production and calcium signaling, and enhanced oxidative stress and apoptosis (Galvan et al., 2017; Eirin et al., 2016).

As is well known, mitochondrial biogenesis needs to meet specific metabolic and energetic cellular demands and this process is regulated through an interconnected set of transcription factors that controlled energy status and cellular adaptive responses. The peroxisome proliferator-activated receptor γ coactivator-1 (PGC-1) is defined as the principal factor regulating mitochondrial biogenesis and energy metabolism (Puigserver et al., 1998; Galvan et al., 2017). Spiegelman et al*.* were the first to discover PGC-1α, and only later, further studies demonstrated a higher expression of PGC-1α in several organs with high energy request such as kidneys and heart. PGC-1α exerts its regulatory effects on mitochondrial function by binding and co-activating several transcription factors such as nuclear respiratory factor-1 (NRF-1), NRF-2, and the estrogen-related receptors (ERR) (Scarpulla, 2008; Baldelli et al., 2013). These interactions mediate the control of some mitochondrial genes involved in mitochondrial biogenesis, lipid oxidation, glycolysis, and ATP synthesis. Moreover, the contribution of these transcriptional factors is cell-type dependent and explains the presence of different metabolic programs in several cell types. Different studies demonstrated that PGC-1α increases mitochondrial content, oxidative phosphorylation, and fatty acid oxidation.

Moreover, the decreased expression of PGC-1α is correlated with a reduced efficiency of mitochondrial biogenesis in AKI (Tran et al., 2011; Lynch et al., 2018) and in CKD (Lim et al., 2012; Galvan et al., 2017; Chung et al., 2018). Indeed, PGC‐1α expression is highly decreased in different preclinical models of AKI, including cisplatin‐induced renal damage (Morigi et al., 2015) (Portilla et al., 2002), folate (Ruiz-Andres et al., 2016), IRI‐ (Portilla et al., 2002; Lempiäinen et al., 2013), and lipopolysaccharide (LPS)‐induced AKI (Tran et al., 2011). In the I/R model, PGC‐1α‐knockout mice underwent worse pathological renal damage with increased fat deposits and clear signs of tubular damage compared to wild‐type (WT) mice (Tran et al., 2016).

Moreover, Ruiz‐Andres et al. (2016) demonstrated that the increase in inflammatory mediators during AKI led to histone deacetylation that enhanced chromatin condensation, reducing accessibility of PGC-1α gene and suppressed its expression. Moreover, Smith et al. (2015) showed that in endotoxemic conditions, LPS systemic exposure promoted the activation of TLR-4 and MAPK/ERK pathway, inducing a significant decrease in PGC‐1α synthesis. Its gene expression is also reduced in various animal models of CKD, such as unilateral ureteral obstruction‐induced fibrosis (Han et al., 2017), db/db diabetic mice (Jianyin et al., 2016; Yuan et al., 2018), and streptozotocin‐induced diabetic mice (Kwon et al., 2017). Indeed, the inactivation of the PGC-1α signaling pathway has been demonstrated as a key mechanism of diabetic nephropathy (Dugan et al., 2013; Platt and Coward, 2017). Until now the role of this gene in renal aging is still unclear. Lim et al. (2012) showed that PGC‐1α expression was decreased in the kidneys of aged mice; moreover, a recent study demonstrated that the activation of SIRT1 and AMPK by an agonist of PPARα, known as fenofibrate, increased the expression of PGC‐1α and ERRα improving mitochondrial dysfunction in aged kidneys (Kim et al., 2016). Therefore, reestablishing PGC‐1α expression could be a promising therapeutic strategy to counteract senescence and progression of CKD (Lee et al., 2019).

The imbalance between mitochondrial fission and fusion has been described in several diseases including cardiovascular and neurodegenerative diseases, diabetes, and cancer (Eirin et al. 2016). Recently, two studies demonstrated increased mitochondrial fragmentation in renal tubular cells in CKD models (Brooks et al., 2009; Zhan et al., 2013). Therefore, there is an urgent need to discover a new treatment that could ameliorate mitochondrial dysfunction in CKD and other settings in which mitochondrial impairment has a key role (Che et al., 2014).

In addition, there are pieces of evidence that mitophagy significantly reduces during physiological aging (Sun et al., 2015; García-Prat et al., 2016). Therefore, the accumulation of damaged mitochondria modifies their functional homeostasis, increasing accumulation of oxygen reactive species (ROS) in aged organs. In several experimental animal models, the deletion of autophagy genes induced the accumulation of dysfunctional mitochondria and strongly increased ROS levels. The administration of antioxidant therapies mitigated these dysfunctional effects; therefore, the decline of autophagy and in particular mitophagy promotes a harmful increase in oxidative stress that also augments age-related tissue injury (Wu et al., 2009; Bratic and Larsson, 2013; Yan and Finkel, 2017) **(** Figure 1 **)**.

Specifically, mitophagy has been implicated in several kidney diseases, including both AKI (Ishihara et al., 2013) and CKD (Namba et al., 2014). After IRI, the expression of BNIP3, a member of Bcl2 family that enhances mitophagy, is strongly reduced in renal tubular cells, suggesting a possible link between mitophagy and renal function. Moreover, the inhibition of mitophagy is also associated with CKD, indicating that a disruption of renal tubular mitochondrial quality control contributes to the pathogenesis of CKD (Tang et al., 2015). These studies imply that target therapies that improve the mitophagy process could induce several beneficial effects in counteracting age-related functional decline. In addition, some natural molecules such as the polyamine spermidine are able to induce both autophagy and mitophagy with an extension of lifespan and recovery in age-associated diseases (Eisenberg et al., 2016).

As is well known the increase in mitochondrial ROS has a strong impact on several pathways involved in apoptosis and senescence process. However, this traditional point of view has recently been changed with a new hypothesis that only the right amount of ROS is necessary to assure physiological cellular function (Eirin et al., 2016). The mutation rate in the mitochondrial genome is caused by a higher amount of ROS, leading to mitochondrial dysfunction, which promotes a further increase in ROS production that in a circle contributes to mtDNA damage. This progressive damage in the mitochondrial genome accelerates the aging process and has been associated with cardiovascular and CKD (Koyano et al., 2014). Generally, the mutations in mtDNA are mostly located in the genes implicated in mtDNA integrity, transcription, and RNA maturation (Bingol et al., 2014).

Therefore, the emerging literature highly supports the critical role of mitochondrial dysfunction in aging process and the development and progression of renal diseases. However, our knowledge of mitochondrial impairment in CKD has yet to be fully clarified. Therefore, additional studies are necessary to better delineate therapeutic strategies for recovery of mitochondrial function in the CKD setting (Eirin et al. 2016).

### Autophagy in Renal Disease and Aging

To date, accumulating evidence has facilitated our understanding of the role of autophagy in kidney physiology, diseases, and aging. Ischemic, toxic, immunological, and oxidative injury can enhance autophagy in proximal tubular cells and podocytes modifying the course of renal diseases (Huber et al., 2012). Autophagy is a highly conserved balancing mechanism for energy and resource; this process helps cells to “self-eat” endogenous material and recycle cellular components for maintaining cellular integrity and energy (Parzych and Klionsky, 2014; De Rechter et al., 2016; Lin et al., 2019). Dysregulation of autophagy is associated with the accumulation of autophagosomes, intracellular damaged proteins, and organelles that contribute to the progressive deterioration of the kidney’s functions (Huber et al., 2012).

As is well known, glomerular podocytes are terminally differentiated cells and their fate is strongly dependent on their capacity to build an efficient response against any insults. Moreover, proximal tubular cells require a large amount of mitochondria to assure protein reabsorption from glomerular filtrate (Christensen and Nielsen, 1991). Therefore, both podocytes and tubular cells have to assure their homeostasis and functions through the autophagy process avoiding the progression of kidney aging and CKD. Indeed, several studies have demonstrated a protective role of autophagy to counteract aging process in podocytes and tubular cells (Hartleben et al., 2010; Kimura et al., 2011; Weide and Huber, 2011).

Both rat and mouse models of renal aging showed that the accumulation of dysfunctional mitochondria and age-associated proteins, such as SQSTM1 (ubiquitin-binding protein p62), was associated with a decrease in autophagy activity (Kume et al., 2010; Cui et al., 2012). Therefore, aging kidneys lose their ability to induce autophagy and accelerate the progression of renal dysfunction.

Furthermore, autophagy-related protein (ATG) ATG5 has a critical role in converting LC3-I to LC3-II, which is crucial for autophagosome formation, and ATG5 knockout mice presented a decrease of autophagy in both podocytes and proximal tubular cells (Hartleben et al., 2010; Kimura et al., 2011). Specifically, podocytes accumulated dysfunctional mitochondria and ubiquitinated proteins aggregate, thereby increasing proteinuria and development of glomerulosclerosis (Hartleben et al., 2010). Also, proximal tubular cells increased abnormal mitochondria, ubiquitinated proteins such as SQSTM1, which significantly increased apoptosis. Therefore, the absence of basal autophagy is strongly correlated to an increase in kidney aging (Figure 1).

Recent findings demonstrated that energy restriction may provide a strategy to preserve the cellular autophagic process. Since calorie restriction induces physiological autophagy, it can counteract age-associated renal damage preventing albuminuria, glomerulosclerosis, and tubulointerstitial lesions (Wiggins et al., 2005; McKiernan et al., 2007; Colman et al., 2009; Kume et al., 2010). Moreover, calorie restriction counteracts hypoxic conditions, restoring autophagy activity and protecting the kidney against aging and CKD (Kume et al., 2010). Kume S. et al. showed in a mouse model of aging kidney that the molecular mechanism underlying the calorie restriction-mediated restoration of autophagy against hypoxia was SIRT-1. SIRT1, as its yeast homolog silent information regulator 2 (Sir2), plays a key role in promoting survival and restoring the autophagy in aging kidney by the deacetylation of FOXO3/FOXO3A, a transcription factor involved in the stress-oxidative pathway (Kume et al., 2010).

In addition to age-associated CKD, several findings demonstrated a major risk of AKI development in elderly population. Recently, numerous studies have demonstrated that the activation of autophagy protects proximal tubular cells from several insults during AKI. Indeed, Lai and colleagues showed in a rat model of IRI an increased expression of ATG proteins in tubular compartment, suggesting the autophagic activity as a key mechanism in renal pathology (Chien et al., 2007; Wu et al., 2009). Despite some controversial studies, emerging evidence has clearly demonstrated a reno-protective role of autophagy in renal tubular compartment during AKI. The insurgence of AKI is strictly related to increasingly aging population that presented a natural decrease of autophagy process. Although further studies are needed, the recovery of autophagic activity in the aging kidney would prevent tubular dysfunction against both acute and chronic damage.

### Epigenetic modifications

Epigenetic mechanisms are crucial in physiological condition and influence the gene expression without permanent modifications in the original DNA sequence (Nangaku et al., 2017); these changes include DNA methylation, histone posttranslational modifications, and miRNA pattern variations (Shiels et al., 2017). Epigenetics has recently been demonstrated to have a fundamental role in the development of CKD (Jones et al., 2015). Many factors, from the physiological to the pathological environment as the uremic toxins, oxidative stress, and inflammation, increase the occurrence of epigenetic changes as well as progression to CKD (Chu et al., 2017; Morgado-Pascual et al., 2018). Also miRNAs are involved in posttranscriptional regulation of gene expression and have been described as potential biomarkers in CKD progression (Zhao et al., 2019). The role of miRNAs in AKI has been also investigated, and several miRNAs have been associated with inflammation and fibrotic process (Lee et al., 2014).

Most of the knowledge about DNA methylation derives from experimental studies in diabetic nephropathy (Morgado-Pascual et al., 2018). DNA methylation patterns in specific genes involved in glucose metabolism or kidney fibrosis are considered potential biomarkers in diabetic nephropathy disease (Morgado-Pascual et al., 2018). Indeed, several experimental studies demonstrated that hypomethylation of the connective tissue growth factor (CTGF) gene promoter is associated with decreased glomerular filtration rate (eGFR) and declined kidney function (Yi et al., 2011; Zhang et al., 2014). In addition, emerging evidence showed that several methylated genes in whole blood samples of diabetic nephropathy subjects correlated to inflammation and apoptosis (Bell et al., 2010; Sapienza et al., 2011).

During kidney transplantation, the procedure itself with the unavoidable IRI, the cold ischemia, and acute rejection can worsen the graft outcome, inducing oxidative stress, inflammation, and vascular complications, which consequently predispose to aberrant DNA methylation changes and the acceleration of kidney aging (Parker et al., 2008; Franzin et al., 2020). Recently, several studies highlighted the relevance of epigenomic, transcriptomic, and proteomic signatures in renal graft that correlated to decline in kidney health and CKD progression (O’Sullivan et al., 2017). Experimental studies of rat and mouse models of IRI and CKD described altered DNA methylation as the crucial process for AKI to CKD transition (Franzin et al., 2020). In the pathological context of the UUO model, Shasha Yin et al. showed that TGFβ decreased Klotho protein expression through Klotho promoter hypermethylation, inducing tubule-interstitium fibrosis. Moreover, in a rat model IRI, Pratt et al. (2006) demonstrated that alterations in C3 methylated promoter region caused an increased activation of the complement system that is strongly involved in inflammation and renal damage.

In addition, aberrant hypermethylation of extracellular matrix laminin pattern genes has been found strongly involved in the development of glomerulosclerosis and tubulointerstitial fibrosis in older kidneys. Bechtel et al. showed that the aberrant methylation of *RASAL1* gene induced an increased activation of Ras-GTPase pathway in fibroblasts, leading to proliferation and fibrosis. Complement activation has been associated with global tubular epithelial cell DNA hypomethylation (Castellano et al., 2019), which was present in premature and accelerated renal aging (Day et al., 2013; Johansson et al., 2013; Quach et al., 2017). Finally, we recently highlighted the role of complement component C5a in promoting DNA hypomethylation of several genes involved in premature senescence pathway, SASP phenotype, and cell cycle arrest (Castellano et al., 2019) in a swine model of IRI. In addition, different studies have found that posttranscriptional histone modifications are involved in experimental renal fibrosis in several pathological conditions such as CKD model, diabetic nephropathy, lupus nephritis, Adriamycin-induced renal damage, and AKI (Morgado-Pascual et al., 2018). Interestingly, there are ongoing clinical trials studying the use of therapeutic approaches in DNA methylation and histone modifications in renal cancer (Morgado-Pascual et al., 2018). All these epigenetic modifications represent a novel field of study to identify new therapeutic strategies and novel biomarkers to overcome the limitations related to the early diagnosis and the prevention of CKD and kidney aging.

### DNA Damage Repair and Kidney Aging

Aging is considered a progressive decline of cell functions, characterized by an insufficiency of response to insults that leads to the accumulation of DNA damage that in turn enhances cell death or the acquirement of a dysfunctional phenotype closely associated with aging processes (Lombard et al., 2005). Accordingly, mice that could not repair DNA damage presented clear signs of premature aging; indeed, young mice with defects in DNA helicase functions presented reduced survival and developed osteoporosis and cachexia, which are identified as aging features (De Boer et al., 2002). Defects in DNA repair in genetic diseases such as Hutchinson–Gilford Progeria, Werner syndrome, Nijmegen breakage syndrome, Fanconi anemia, and Bloom syndrome increased the development of premature aging (Fumagalli et al., 2014; Pan et al., 2016; Li et al., 2016; D’Alessandro et al., 2018). As is well known, DNA repair pathways include base excision repair (BER), nucleotide excision repair (NER), mismatch repair (MMR), and double-strand break repair (DSBR) (Maynard et al., 2015). DSBR is specific for repairing DSBs, mainly by either error-prone rejoining of the broken DNA ends (nonhomologous end joining [NHEJ]) or accurately repairing the DSB using information on the undamaged sister chromatid (homologous recombination [HR]) (Wyman and Kanaar, 2006; Iyama and Wilson, 2013) However, not all DNA modifications are associated with the aging process. For instance, some defects in MMR are involved in tumorigenesis and do not directly trigger senescence process (Papadopoulos and Lindblom., 1997). Otherwise, defects in other DNA repair mechanisms such as BER, NER, NHEJ, and HR are associated with aging-related diseases. For example, alterations in mitochondrial BER induced a decreased activity of four enzymes, DNA glycosylase, endonuclease, DNA polymerase, and DNA ligase that are involved in DNA repair, promoting aging process (Gorbunova et al., 2007; Maynard et al., 2009; Boesch et al., 2011). In addition, other studies showed that a reduced NHEJ was present in neurons from old rats. Telomere maintenance is another important way to assure genome stability and deficiency in this process is associated with cellular senescence and aging (Rodier et al., 2005). Therefore, the increase in DNA damage or the deficiency in DNA repair enhances several age-associated diseases (Pan et al., 2016).

Many features involved in AKI predispose patients to develop CKD, with an incidence of 10% in the world’s population (Bonventre and Yang, 2011; Kishi et al., 2019). Several studies showed a maladaptive response of tubular cells during AKI, characterized by the occurrence of modifications in DDR that could accelerate the aging process and the development of CKD (Ferenbach and Bonventre, 2016; Yang et al., 2010; Grgic et al., 2012). However, the recent literature has not yet demonstrated a direct correlation between DDR and specific renal compartment injury and long-term consequences in renal function (Kishi et al., 2019). Several studies defined DNA damage as a hallmark of several forms of renal damage (Ma et al., 2014; Pabla et al., 2008; Zhu et al., 2015); following DNA strand break (Figure 1), several sensor kinases such as ataxia telangiectasia mutated (ATM), ATM- and Rad3-related (ATR), and DNA-dependent protein kinase (DNA-PK) are activated and in turn enhance the activation of checkpoint kinases 1 and 2 (Chk1 and Chk2), which control cell-cycle progression through the G/S or G/M checkpoint (Brown and Baltimore, 2003; Sancar et al., 2004; Kidiyoor et al., 2016; Li et al., 2016). The DDR process is activated in response to IRI and ATP depletion (Ma et al., 2014). However, when DNA damage is severe, the DDR process is not sufficient to repair DNA and to overcome the insult; accordingly with other studies, we have recently shown that tubular cells arrested cell cycle and acquired a dysfunctional phenotype, known as SASP (Castellano et al., 2019), resulting in the generation of pro-inflammatory and pro-fibrotic cytokines, which promote the development of kidney fibrosis and the progression to CKD (Yang et al., 2010; Grgic et al., 2012).

Studies in mouse models with the deletion of the ATR gene led to an increase in DNA damage, alterations in tissue homeostasis, and the rapid development of age-related phenotypes (Ruzankina et al., 2007; Murga et al., 2009; McKinnon, 2009). Interestingly, cisplatin-stimulated renal tubular cells activated ATR gene (Pabla et al., 2008), but its contribution to the maladaptive response in these cells has to be clarified. The role of DDR in the aging process and in the progression of CKD cannot be ignored (Wang et al., 2017). Many factors that are involved in CKD can cause alterations in DDR response and accelerate aging process in renal parenchyma and, immune cells, endothelial cells, and progenitor and stem cells (Tasanarong et al., 2013; Goligorsky, 2014; Klinkhammer et al., 2014; Bonventre,; Bautista-Niño et al., 2016).

As described previously, when these cells become senescent, they are metabolically active and have a distinct secretome termed SASP (Coppé et al., 2008). The acquirement of SASP is accompanied by genomic damage and epigenetic abnormalities (Castellano et al., 2019). The principal causes include radiation agents, cytotoxic drugs, topoisomerase inhibitors, ROS accumulation, and other agents (Hayflick, 1965; Larsson, 2011; Demaria et al., 2017). These stimuli provoke single- or double-strand breaks in DNA and DDR response cannot recover DNA damage (Campisi, 2013; Johmura et al., 2016). The increase and persistence of DNA damage extended cycle arrest in G1 and G2 phases by the activation of the DDR pathway (Malaquin et al., 2015). Therefore, DDR promotes a prolonged cell cycle arrest through the accumulation and activation of cyclin-dependent kinase inhibitors (CKIs) such as tumor protein p53 (TP53 or p53), p21CIP1 (p21), and p16INK4a (p16) (El-Deiry et al., 1993; Wade Harper et al., 1993). These CKIs inactivate cyclin-dependent kinases (CDKs), which are necessary for the activation and progression of cell cycle. Therefore, CDKs are not able to mediate the phosphorylation of the retinoblastoma tumor suppressor (Rb), which in turn cannot activate E2F protein complex, arresting G1/S progression and DNA replication, or G2/M transition and mitosis, avoiding cellular capability of proliferation and inducing senescent phenotype. In other cases, some oncogenes, such as H-RAS, the mitogen-activated protein kinase (MAPK) signaling pathway, and tumor suppressors p53 and retinoblastoma (Rb), are strongly activated, inducing additional alterations in DNA sequences and contributing to cellular senescence (Larsson, 2011; Campisi, 2013).

Under certain conditions, in the absence of DNA damage, DDR response can be activated promoting the acquisition of senescent phenotype. Indeed, the DDR arrests cell cycle through the activation of the p53/p21 and p16INK4a/ Rb signaling, induces senescence growth-arrested state, and maintains SASP in senescent cells (Acosta et al., 2008; Kuilman et al., 2008; Campisi, 2013). The synthesis and release of SASP factors is associated with the activation of the transcription factors NF-κB and CCAAT enhancer binding protein β (C/EBPβ) (Acosta et al., 2008; Kuilman et al., 2008). Thus, both DDR response and NF-κB activation significantly contribute to establishing and maintaining SASP condition. Taken together, the increase in SASP exacerbates DDR and influences senescent cells to produce and release more senescent secretome, contributing to functional deterioration of neighboring cells and accelerating the process of cellular aging.

## Potential Biomarkers of Renal Aging: Extracellular Vesicles

Extracellular vesicles (EVs) have recently been recognized as important vehicles of intercellular communication in both physiological and pathological states, as renal fibrosis (Johnstone et al., 1987; Angus et al., 2004; Camussi et al., 2010; Mathivanan et al., 2010; Jing et al., 2019). A mountain of reliable evidence has attributed to the EVs a proven role in tissue maintenance and repair (Panagiotou et al., 2016). These vesicles are synthesized and secreted by several cell types and are released in different body fluids from plasma to bronchial lavage fluid (Buzas et al., 2014; Konala et al., 2016). EVs shuttle biological molecules such as several proteins, lipids, DNA fragments (Thakur et al., 2014), mRNA, micro-RNAs (miRNAs), and other noncoding RNAs that could modify cellular microenvironment in physiological and pathological conditions affecting cellular behavior (Valadi et al., 2007).

Besides their role in modulating cell response, they have recently been identified as new biomarkers of both cancer and aging (Rak, 2013; Machida et al., 2015). Indeed, tumor cells are able to release EVs that carry pathological information such as oncogenic molecules and RNA and miRNA implicated in drug resistance (Rak, 2013). EVs derived from saliva or serum could contain several miRNA involved in aging disease (Machida et al., 2015). In general, EVs play a key role in both physiological and pathological conditions associated with the senescence process (Desdín-Micó and Mittelbrunn, 2017). Therefore, EVs can exert beneficial effects promoting tissue regeneration or detrimental effects under pathological conditions such as oncogenesis and aging-related disease (Yang and Robbins, 2011; Urbanelli et al., 2016).

In addition, several studies have demonstrated that endogenous or exogenous stress stimuli induce an increase in specific EVs in the cellular cytoplasm that, when secreted, can enable other cells to better prepare a successful response (Urbanelli et al., 2016). However, in long term the increase in these EVs can also promote a maladaptive response and can accelerate aging process (Shiels et al., 2017). The presence of senescent cells induces also a release of EVs that modify cellular microenvironment and influence neighboring nondamaged cells to, in turn, acquire senescent phenotype (Olivieri et al., 2015; Biran et al., 2017; Robbins, 2017). Therefore, EVs secreted by senescent cells are defined as noncanonical part of the SASP and are able to trigger both physiological and pathological aging (Olivieri et al., 2015; Biran et al., 2017; Robbins, 2017). Indeed, senescence-associated EVs have been described in age-associated lung diseases; specifically, the miR-21 found in exosomes has been associated with idiopathic pulmonary fibrosis (Kadota et al., 2018).

Recently, several works showed the potential role of EVs in renal diseases (Bruno et al., 2016; Karpman et al., 2017). The direct involvement of EVs in renal aging is still an undiscovered field. However, mounting studies revealed a strong association between EVs and the progression of renal fibrosis, which is defined as the end stage of all chronic kidney diseases. Since cellular senescence is involved in kidney fibrosis and renal disease progression, we can speculate that most of EVs analyzed could be part of the SASP.

Different studies have shown that EVs’ secretion and content could be influenced by several stress conditions such as ROS increase, hypoxic state, and pH alteration (Parolini et al., 2009; Hedlund et al., 2011; de Jong et al., 2012; King et al., 2012). For example, under hypoxic conditions exosomes released by damaged tubular epithelial cells contain TGF-β1 mRNA and transfer it to adjacent fibroblasts that synthesized and secreted TGF-β1 protein; the increase in TGF-β1 induces pleiotropic effects on neighboring fibroblasts that started to proliferate and release several components of extracellular matrix-promoting fibrosis (De Wever and Mareel, 2003; Borges et al., 2013); also high glucose environment induces glomerular endothelial cells to secrete exosomes containing TGF-β1, thereby promoting renal interstitial fibrosis (Wu et al., 2016). Therefore, the involvement of EVs in the TGF-β pathway and fibrosis has been considered an important mediator in the progression of renal diseases.

Recently, Wnt/β-catenin signaling has been observed in several diseases with renal fibrosis and upregulated in aging kidney (Surendran et al., 2005; Castellano et al., 2019; Miao et al., 2019). Recent studies have demonstrated that exosomes derived from cells that acquired myofibroblast phenotype via EMT increase the expression of β-catenin and activate the canonical Wnt/β-catenin signaling in neighboring cells (Wu et al., 2017). Therefore, the inhibition of Wnt/β-catenin signaling could slow the onset of age-related mitochondrial dysfunction and renal fibrosis in several renal diseases (148). Recently, the treatment of CKD to prevent kidney fibrosis and terminal end-stage renal disease by inhibiting EVs has attracted considerable attention (Lv et al., 2018).

Other studies have suggested a potential role of EVs in genomic instability and aging process, through the transfer of retrotransposons (Kawamura et al., 2017). Retrotransposons are mobile DNA elements that generate several copies of themselves into the host DNA (De Cecco et al., 2013). The process of retrotransposition or an unsuccessful transposition event can induce genomic instability and mutagenic events. De Cecco M. et al. have observed that the transfer of retrotransposons is increased in senescent cells and could be a driving force in aging process (De Cecco et al., 2013).

As previously described, EVs can exert beneficial effects promoting tissue repair; in particular, EVs derived from stem cells and regulatory cell types showed significant regenerative properties in several diseases (Merchant et al., 2017). Several studies demonstrated a therapeutic effect in cases of cardiac, lung, retinal, neural, pancreatic, and kidney damage. Moreover, there is a growing interest to explore the EVs capable of reverting aging process and organ injury. In the field of renal recovery after insults or age-related diseases, EVs derived from mesenchymal stem cells could counteract acute and chronic injury by inhibiting apoptosis and promoting regeneration of parenchymal damaged cells (Tetta et al., 2011; Biancone et al., 2012).

In addition, microvescicles from endothelial progenitor cells also showed therapeutic potential in preventing acute kidney failure after IRI in rats. Cantaluppi V. et al. demonstrated that endothelial EVs exerted their protective functions through specific miRNA that were delivered to resident renal cells for kidney function recovery (Cantaluppi et al., 2012). Therefore, we think that studying the role of EVs in the context of physiological and pathological aging will increase our knowledge on the principal mechanisms that could be targeted to ameliorate organ functions in several diseases.

## Therapeutic Intervention: The Seno-Therapeutic Drugs

In the last few years, the development of compounds able to directly eliminate senescence cells or to target the effects of senescent cells has found a vivid interest in the complex field of age-related pathologies. Seno-therapeutic agents hold promise for the utilization in treating disorders related to senescent cell accumulation such as neurodegenerative diseases, atherosclerosis, cancers, kidney injury, atherosclerosis, chronic obstructive lung disease, idiopathic pulmonary fibrosis, diabetes, as well as complications of organ transplantation, radiation, and chemotherapy (Tchkonia et al., 2013; Palmer et al., 2015) (Figure 1).

As already discussed, senescent cell viability is strictly dependent on apoptosis resistance and anti-apoptotic signaling thus leading researchers in nephrology to extend the application of therapeutic strategies adopted in oncology also to prevent the complications of kidney aging.

In particular, senescent cells rely on several survival pathways, including those regulated by BCL‐2/BCL‐XL‐family, PI3K/AKT‐, p53/p21/serpine‐, HIF‐1α‐components to confer resistance to their pro‐apoptotic SASP and intracellular cell damage signals (Pan et al., 2017; Knoppert et al., 2019; Docherty et al., 2020).

The “seno-therapeutic drugs” are an umbrella term that includes different molecules as the senolytics (compounds that kill senescent cells selectively), senomorphics (i.e., molecules that can inhibit SASP, modulate functions and morphology of senescence cells, or delay the progression of young cells to senescent cells), and senoinflammatory mediators (that are immune-system effectors of the clearance of senescent cells) (Kim and Kim, 2019).

For the discovery of these compounds with senotherapeutic properties, the production of transgenic animals has been combined with cutting-edge. Despite their complexity, all the approaches shared the common feature that survival of senescent cells is dependent on specific genes implicated in pro-survival and anti-apoptotic pathways and that these signaling could be targeted to facilitate selective clearance of senescent cells without affecting normal cells (Knoppert et al., 2019; Table 1). In addition, it became evident that senolytics described so far are limited in the senescent cell types they are able to target (Zhu et al., 2016). Here, we will discuss the experimental approaches that target the p16INK4a, BCL-2/BCL-xl/BCL-w, and p53 survival pathway of senescent cells.

**TABLE 1:** Senolytic and senomorphic drugs.

Agents	Function	References	Study design	Therapeutic field	Major findings
Navitoclax (ABT263)	Inhibitor of BCL-2 and BCL-xL				
*In vitro* studies and animal model					
		Chang et al. (2016)	Animal model: oral administration of ABT263 to either sublethal irradiated or normally aged mice	Aged tissue stem cells	Increased hematopoietic and muscle stem cell function
		Zhu et al. (2016)	*In vitro* study: induction of cellular senescence in HUVECs, IMR90 cells, and preadipocytes	Senescent cells	Reduced viability of senescent HUVECs, and IMR90 cells
		Pan et al. (2017)	Animal model: mice model of ionizing radiation–induced pulmonary fibrosis	Chronic lung fibrosis	Reduced viability of senescent type II pneumocytes and decreased pulmonary fibrosis
Clinical studies					
		Gandhi et al. (2011)	NCT00445198: interventional study (phase I/II) with 86 participants with small-cell lung cancer (SCLC) or other nonhematological malignancies	Small-cell lung cancer (SCLC) or other nonhematological malignancies resistant to chemotherapy-induced apoptosis	Phase I results: safety and toleration dose
		Wilson et al. (2010)	NCT00406809: interventional study (phase I/II) with 81 participants with relapsed or refractory lymphoid malignancies	Relapsed or refractory lymphoid malignancies	Phase I results: safety and toleration dose
		Roberts et al. (2015)	NCT00788684: interventional study (phase I) with 29 participants with CD20-positive lymphoid malignancies	Lymphoid tumors	Phase I results: safety dose in combination with rituximab
			NCT01989585: interventional study (phase I/II) with 75 participants with BRAF mutant melanoma or solid tumors that are metastatic	BRAF mutant melanoma or solid tumors that are metastatic	NCT01989585: ongoing study (primary completion date: December 31, 2021)
			NCT03366103: interventional study (phase I/II) with 79 participants with relapsed small-cell lung cancer and other solid tumors	Relapsed small-cell lung cancer and other solid tumors	NCT03366103: ongoing study (estimated study completion date: August 31, 2021)
Quercitin	Antioxidant activity and inhibitor of PI3K/AKT and p53/p21/serpines				
*In vitro* studies and animal model					
		Kim et al. (2019)	Animal model: C57BL/6 J mice fed high-fat diet	Renal dysfunction in dyslipidemia and obesity setting	Amelioration of obesity-induced renal senescence
Quercitin + dasatinib	Antioxidant activity and inhibitor of PI3K-AKT and p53, p21, serpines, and tyrosine kinase inhibitor				
*In vitro* studies and animal model					
		Zhu et al. (2015)	*In vitro* study: senescent preadipocytes and HUVECs	Aging and radiation damage	*In vitro*: reduced viability of senescent preadipocytes and HUVECs
			Animal model: aging C57Bl/6 mice with or without radiation		In vivo: extension of lifespan, amelioration of cardiovascular function, and reduced radiation injury
		Xu et al. (2018)	Animal model: transplantation of senescent cells into young mice	Aging‐related disease	Extension of lifespan and amelioration of senescent cell-induced physical dysfunction
		Wang et al. (2019)	Animal model: female C3H mice and male Sprague–Dawley rats with radiation ulcers	Aging and radiation ulcers	Elimination of senescent cells in radiation ulcers
		Palmer et al. (2019)	Animal model: obese mice	Obesity‐induced metabolic dysfunction	Decrease of metabolic and adipose tissue dysfunction
Clinical studies					
		Justice et al. (2019)	NCT02874989: open-label human pilot study in idiopathic pulmonary fibrosis with 26 participants	Idiopathic pulmonary fibrosis	Reduced pulmonary fibrosis
		Hickson et al. (2019)	NCT02848131: open-label phase 1 pilot study with diabetic kidney disease in 16 participants	Chronic kidney disease	Reduced adipose tissue senescent cells, skin senescent cells, and circulating SASP factors
Quercetin + resveratrol	Antioxidant activity and inhibitor of PI3K-AKT and p53, p21, and serpines				
*In vitro* studies and animal model					
		Abharzanjani et al. (2017)	*In vitro* study: human embryonic kidney cell (HEK-293) cultured in high-glucose conditions	Hyperglycemia and diabetic nephropathy	Increased expression levels of antioxidants and reduced aging markers in HEK cells in hyperglycemic conditions
JAK inhibitor (ruxolitinib)	Inhibitor of JAK (janus kinase) pathway				
*In vitro* studies and animal model					
		Xu et al. (2018)	Animal model: old C57BL/6 male mice	Aging‐related disease	Reduced inflammation and alleviated frailty in aged mice
			*In vitro* study: preadipocytes and HUVECs		
NBD peptide	Inhibitor of IKK/NFB pathway				
*In vitro* studies and animal model					
		Tilstra et al. (2012)	Animal model: progeroid model mice	XFE progeroid syndrome	Reduced oxidative DNA damage and stress and delayed cellular senescence
KU-60019	Inhibitor of ataxia-telangiectasia mutated (ATM) kinase				
*In vitro* studies and animal model					
		Kang et al. (2017)	*In vitro* study: human diploid fibroblasts and ATM-deficient fibroblasts	Aging‐related disease	*In vitro*: functional recovery of thelysosome/autophagy system, mitochondrial functional recovery, and metabolic reprogramming
			Animal model: wound healing assay in old C57BL/6 J mice		*In vivo*: accelerated
JH4	Interfering binding of progerin and lamin				
*In vitro* studies and animal model					
		(Lee et al., 2014)	Animal model: HGPS-progeroid mice	Hutchinson–Gilford progeria syndrome and aging disease	Reduced nuclear deformation and senescence process
					Extension of lifespan in the HGPS-progeroid mice
Juglanin	Not reported				
*In vitro* studies and animal model					
		Yang et al. (2014a)	*In vitro* study: adriamycin-induced human dermal fibroblast (HDF) senescence	Tissue repair and regeneration	Decreased senescence in HDFs
Quercetin-3-O-β-D-glucuronide	Not reported				
*In vitro* studies and animal model					
		Yang et al. (2014b)	Animal model: adriamycin-induced HDF and HUVEC senescence	Tissue repair and regeneration	Decreased senescence in HDFs and HUVECs
Loliolide	Not reported				
*In vitro* studies and animal model					
		Yang et al. (2015a)	Animal model: adriamycin-induced HDF and HUVEC senescence	Tissue repair and regeneration	Decreased senescence in HDFs and HUVECs
Quercetagetin 3,4′-dimethyl ether	Not reported				
*In vitro* studies and animal model					
		Yang et al. (2015b)	*In vitro* study: adriamycin-induced HUVEC senescence	Tissue repair and regeneration	Decreased senescence in HUVECs
Rapamycin	Inhibitor of mTOR kinase				
*In vitro* studies and animal model					
		Antonioli et al.( 2019)	*In vitro* study: primary human bone marrow (BM) MSC samples of five healthy young adults	Tissue engineering and cell-based therapies	Retard senescence and extend stemness properties
		Chen et al. (2009)	Animal model: old C57BL/6 wild-type mice	Aging hematopoietic stem cells	Increased mice lifespan, self-renewal of hematopoietic stem cell, enabled vaccination
		Arriola Apelo et al. (2016)	Animal model: old female C57BL/6 J mice	Aging‐related disease	Extension of mice lifespan
		Wang et al. (2017)	Animal model: old WT and Nrf2 knockout mice	Aging‐related disease	Inhibition of the secretory phenotype of senescent cells
		Lesniewski et al. (2017)	Animal model: old male B6D2F1 mice	Aging‐related disease	Improvement of arterial function, reduced oxidative stress, AMPK activation, and increased expression of proteins involved in the control of the cell cycle
RAD001 (analog of rapamycin)	Inhibitor of mTOR kinase				
*In vitro* studies and animal model					
		Shavlakadze et al. (2018)	Animal model: old rats	Aging‐related disease	Modulation of age-regulated genes expression in the kidney and liver
Clinical studies					
		Mannick et al. (2014)	Clinical study: 218 elderly volunteers ≥65 years of age	Aging-related disease	Amelioration of immuno-senescence to influenza vaccination
Metformin	Inhibition of NF-kB signaling and Nrf2 modulation				
*In vitro* studies and animal model					
		Fang et al. (2018)	*In vitro* study: human diploid fibroblasts (HDF) and human mesenchymal stem cells (HMSCs)	Aging‐related disease	Amelioration of cellular aging
	Not reported	(Park and shin 2017)	*In vitro* study: primary dermal fibroblasts derived from Hutchinson–Gilford progeria syndrome	Hutchinson–Gilford progeria syndrome	Amelioration of cellular aging
			Animal model: aged BALB/c mice		Reduction of ROS, γ-H2AX foci, and ATM
Clinical studies					
	Not reported	Not reported	Clinical trial: NCT02432287: 16 participants (older adults with impaired glucose tolerance (IGT))	Aging‐related disease	Not reported
	Not reported	Barzilai et al. (2016)	Clinical study: TAME study: enrollment of 3,000 subjects, ages 65–79 years, in ∼14 lefts across the United States	Aging‐related disease (cardiovascular events, cancer, dementia, and mortality)	Ongoing study (recruitment started 2020)

Table summarizing the senotherapies recently discovered, with the indication of the model, type of disease, clinical trials, and references.

### The Clearance of p16Ink4a-Positive Cells to Delay Aging

The importance of p16INK4a signaling to arrest cell cycle in G1/S checkpoint has already been described. Of interest, the number of p16-positive cells increased in both physiological and pathological aging (Valentijn et al., 2018).

In recent seminal studies, Baker et al. (2011); Baker et al. (2016) demonstrated the safety and efficacy of senescent cell depletion *in vivo*. The authors generated transgenic mice (INK-ATTAC) where the senescence biomarker p16INK4a promoter was associated with a “suicide” gene triggering caspase-dependent apoptosis (Baker et al., 2011). A recent improvement of this technology allowed the selective elimination of p16INK4a-positive cells by the administration of the AP20187 molecule. The treatment of transgenic mice with AP20187 led to increased lifespan, providing strong evidence that senescent cell depletion increases healthy lifespan by postponing the onset of several age-associated pathologies. In addition, the clearance of p16Ink4a-positive cells delayed tumorigenesis and attenuated age-related deterioration of several organs, including kidney, where clearance preserved the functionality of glomeruli, reduced glomerulosclerosis, and improved BUN. Importantly, such benefits were not correlated with increased cancer incidence (Baker et al., 2016).

### Targeting BCL-2 Pathway: Navitoclax (ABT263)

The resistance of senescent cells to apoptosis relies on pro-survival BCL-2, BCL-xl, and BCL-w proteins activation. The Bcl-2 family consists of many evolutionarily conserved proteins that share Bcl-2 homology (BH3) domains. During senescence, increased Bcl-2 expression acts as a disruptor of the interaction between the pro-apoptotic proteins Bad and Bax, which cannot form oligomers, inhibiting the mitochondrial outer membrane permeabilization that normally leads to the intrinsic pathway of apoptosis. This property of senescent cells has been used in the establishment of BH3 mimetics, which is able to bind and control different BCL-2 family members.

In an elegant study, Chang et al. (2016) identified Navitoclax (ABT263), a specific inhibitor of BCL-2 and BCL-xL, as a potent senolytic drug able to selectively kill senescent cells in culture by inducing apoptosis. Oral administration of ABT263 to normally aged mice effectively depleted senescent cells, at level of both bone marrow hematopoietic stem cells and muscle stem cells. More importantly, Navitoclax was shown to reduce viability of senescent human umbilical vein epithelial cells, IMR90 human lung fibroblasts, and murine embryonic fibroblasts, but not preadipocytes isolated from lean kidney transplant donors suggesting mechanisms of resistance in obese patients (Zhu et al., 2016).

The great opportunity represented by Navitoclax therapy in chronic fibrosis has recently been documented by Pan et al. in 2017. In a mice model of ionizing radiation-induced pulmonary fibrosis the authors demonstrated that chronic pulmonary fibrosis could be reversed by Navitoclax after it became a progressive disease and offered a new strategy also in other contexts of irreversible fibrosis (Pan et al., 2017). Currently, Navitoclax is under evaluation in several trials to overcome the chemotherapy-induced apoptosis resistance in solid tumors (i.e., melanoma NCT01989585, small-cell lung cancer NCT03366103) and lymphoid tumors (Wilson et al., 2010; Gandhi et al., 2011; Roberts et al., 2015). Despite encouraging results, the use of Navitoclax has been associated with several adverse effects; the most serious are thrombocytopenia due to the effect of the drug on apoptosis in circulating platelets.

### Targeting Senescent Cells by Flavonoids

Other senolytics drugs were recently discovered from the natural product from the class of flavonoids (Panche et al., 2016). Like most flavonoids, quercetin is a polyphenol with potent antioxidant activity, abundant in fruits and vegetables (García-Barrado et al., 2020). Quercetin is involved in several biological functions such as cancer prevention, antiviral activity (Wang et al., 2019; Mrityunjaya et al., 2020), and selectively clearing senescent cells, inhibiting PI3K/AKT and p53/p21/serpines and inducing apoptosis (Russo et al., 2014; Myrianthopoulos et al., 2019). Quercetin has an important anti-oxidative effect, since it is able to target ROS. In addition, quercetin can affect also Nrf2 pathway, which is particularly important in senescent cells. Recently, quercetin has been linked to SIRT1, a NAD deacetylase with important antiaging effect mediated by Klotho, p53, and mitochondrial dysfunction modulation (Wakino et al., 2015). The pivotal role of Klotho signaling as renoprotective function in tubular cells has been well described elsewhere. (Dalton et al., 2017; Wang et al., 2018). Quercetin has been investigated in combination with a tyrosine kinase inhibitor called dasatinib. Several pieces of evidence demonstrated that quercetin and dasatinib, alone and in combination, cause apoptosis in senescent cells without significant effects in quiescent or proliferating cells (Zhu et al., 2015; Xu et al., 2018).

*In vivo*, this combination delays natural aging and age-related symptoms such as obesity disease, atherosclerosis, pulmonary fibrosis, and transplantation of senescent cells. In an elegant study, Xu et al. provide evidence that senescent cells can induce physical dysfunction and decreased survival even in young mice, whereas senolytics enhanced health and increased lifespan in old mice (Xu et al., 2018). The authors demonstrated that transplantation of a few of senescent cells into young mice not only triggered physical dysfunction, but also propagated senescence to other tissues. The transplantation of fewer senescent cells in older recipients further decreased lifespan. In the same study, in explants of human adipose tissue, the combination of dasatinib plus quercetin appeared effective in reducing the number of naturally occurring senescent cells and their secretion of frailty-related pro-inflammatory cytokines. Finally, the authors also showed a protective effect of senolytics when orally administered intermittently, showing a reduction in mortality hazard to 65% (Xu et al., 2018).

Besides the murine model of physiological aging, the combination of quercetin plus dasanitib has been associated with reduction of obesity-induced metabolic dysfunction and recently, in mitigated radiation ulcers (Wang et al., 2019). Indeed, senescent cells accumulate in adipose tissue of obese and diabetic humans and mice (Schafer et al., 2017; Sierra-Ramirez et al., 2020); however, their role is not well understood. For instance, components of the SASP secreted by adipose‐derived senescent cells have been postulated to confer insulin resistance upon metabolic tissues, inhibit adipogenesis, and attract immune cells that can exacerbate insulin resistance (Palmer et al., 2019). In a recent study, Palmer et al. demonstrated that quercetin plus dasanitib treatment in obese mice improved insulin sensitivity, lowered circulating inflammatory mediators, and promoted adipogenesis. More interestingly, also the renal function, one of the most important complications of type 2 diabetes, was significantly improved by microalbuminuria and podocyte function restoration (Palmer et al., 2015; Palmer et al., 2019).

Interestingly, Kim et al. (2019) demonstrated that obesity and dyslipidemia induced renal senescence that was modulated by quercetin treatment. In particular, the authors found that mice fed with high-fat diets showed impaired renal function, cortical oxygenation, and presented glomerulomegaly. Obese hypercholesterolemic mice displayed augmented level of p16, p19, and p53 expression, whereas in quercetin-treated mice senescence was significantly modulated. Quercetin treatment also increased renal cortical oxygenation and decreased plasma creatinine levels in obese mice.

The senolytic effect of quercetin has been analyzed in combination with other compounds such as resveratrol in human kidney cell culture under hyperglycemia condition. Resveratrol is a polyphenol of the stilbene class and is abundant in foods such as wine and fruit. This natural compound has been involved in cancer chemoprevention, protection against cardiovascular diseases, and anti-inflammatory activity. The molecule interferes with several critical pathways such as those of NF-kB, IGF-1R/Akt/Wnt, and PI3K and more importantly can target SIRT1, a member of the class III histone deacetylases (Abharzanjani et al., 2017), strongly involved in kidney functionality and aging (Zhuo et al., 2011; Ugur et al., 2015).

The quercetin and resveratrol were associated with increased expression levels of antioxidants and can reduce aging markers in embryonic kidney cell line in hyperglycemia conditions; furthermore, the gene expression of Sirtuin 1 and thioredoxin (Trx) in all treated groups in comparison with control group increased in a dose-dependent fashion (Abharzanjani et al., 2017).

Recently, these results have been translated in a clinical trial. In the first, the combination of quercetin plus dasanitib was associated with improved physical function in patients with idiopathic pulmonary fibrosis (IPF), a fatal senescence-associated disease (Justice et al., 2019). Last, the same cocktail was investigated in an open label phase 1 pilot study, in which the two drugs were administered for 3 days in subjects with diabetic kidney disease. The authors showed a reduced number of adipose tissue senescent cells, with overall decreases in p16INK4A- and p21CIP1-expressing cells. Interestingly, also adipose tissue macrophages, which are attracted, anchored, and activated by senescent cells, and presented crown-like structures were decreased. Skin epidermal p16INK4A+ and p21CIP1+ cells were reduced, as were circulating SASP factors, including IL-1α, IL-6, and MMPs-9 and MMP-12 (NCT02848131) (Hickson et al., 2019). Moreover, quercetin appears as a promising senolytic treatment in renal aging, also because it is much more selective for senescent endothelial cells during oxidative stress.

### Senomorphics

Besides interventions that directly target senescent cells, in the armamentarium of senotherapeutics other agents offer new strategies for the modulation of senescent cells induced SASP. These senostatic/senomorphic drugs include not only molecules such as Janus kinase (JAK)/STAT pathway inhibitors, inhibitors of NF-kB, and other compounds, but also the caloric restriction diets, sirtuin activators, antioxidants, anti-inflammatory agents, autophagy activators, and mTOR inhibitors. Here, we will review rapamycin and metformin given their key role in preventing kidney aging during CKD.

#### Rapamycin

Rapamycin is a selective inhibitor of the mTOR kinase, a signaling molecule implied in a large plethora of biological functions from cell proliferation and survival, aging, autophagy, and oxidative stress regulation (Kennedy and Lamming, 2016). First isolated from Streptomyces strains, rapamycin is a macrolide acting as an allosteric ligand for mTOR and it is mainly used in clinical practice as an immunosuppressant to prevent rejection of kidney and liver transplants, in the treatment of autoimmune disorders (Stallone et al., 2014; Stallone et al., 2016), at high doses in certain types of cancers such as renal carcinoma and lymphangioleiomyomatosis, a rare and progressive lung disease (Li et al., 2014). Impaired activity of mTOR complexes (mTORC1/mTORC2), particularly mTORC1 overactivation, has been implicated in a wide range of age-related disorders, including human renal diseases. This can be explained by the fact that senescent cells, despite the apparent decline in their proliferative potential, are highly metabolically active cells that acquire a more glycolytic state even in the presence of high oxygen levels, similarly to cancer cells (Sabbatinelli et al., 2019). For that reason, the survival of senescent cells is strictly dependent on mTOR activation; for kidney cells, in particular, the survival is more dependent on mTORC1 than on mTORC2 (Stallone et al., 2015).

*In vitro* experiments, rapamycin was demonstrated to counteractreplicative senescence in reducing the levels of secreted IL6, the major SASP cytokine, and to increase levels of the stem cells marker NANOG (Antonioli et al., 2019). These results confirmed previous findings in which rapamycin increased mice’s life span, restored the self-renewal of hematopoietic stem cells, and enabled effective vaccination against a lethal challenge with influenza virus (Chen et al., 2009); moreover, rapamycin extend both lifespan and healthspan in different animal models (Arriola Apelo et al., 2016), counteracting the replicative senescence in rodent embryonic cells (Pospelova et al., 2012). In addition, rapamycin activity on senescence is correlated with suppression of SASP and this result is mediated by an Nrf2 (nuclear factor E2–related factor 2)-independent mechanism (Wang et al., 2017). Interestingly, the activity of rapamycin against SASP has been also shown to correlate with the downregulation of IL-6 but most importantly with the inhibition of IL-1α translation (Laberge et al., 2015). In a recent model of age-related arterial dysfunction, Lesniewski LA et al. demonstrated that rapamycin treatment reduced oxidative stress, AMPK activation and controlled the cell cycle progression (Lesniewski et al., 2017).

Despite these promising results, concerning health human aging the utilization of rapamacyin is at early stages. The most relevant problem is the serious side effects mainly correlated with immune system suppression (i.e., leucopenia), insulin resistance, dyslipidemia with glomerular dysfunction frequently observed (Powell et al., 2012; Nguyen et al., 2019).

In 2014, a clinical trial in elderly subjects demonstrated that a rapalog (RAD001) ameliorated immuno-senescence, and thereby improved the response of elderly humans to influenza vaccination (Mannick et al., 2014). In this case, the intervention used relatively low or intermittent doses of the pharmacologic agent, and lasted only 6 weeks. Based on this study in patients, Shavlakadze et al. translated the same dose and duration of the treatment in a mouse model of aging in rats (Shavlakadze et al., 2018); by using rats aged 22.5 months (approximately comparable to a 60-year-old person), the authors evaluated the outcome of an intermittent administration of RAD001S (at a dose of 1 mg/kg once a week for 6 weeks) and assessed the transcriptional profiles of kidney, liver, skeletal muscle between young and old rats. The authors found an impressive modulation of age-regulated gene expression (about 37%) in the kidney, mainly associated with inflammation and fibrosis, together with a reduction in the severity of nephropathy lesions in aged rats. Strikingly, RAD001 was well tolerated by old rats, with no significant changes in fasting blood glucose and body weight trajectories compared to controls (Di Francesco et al., 2018).

These findings reinforce the idea that selective mTORC1 inhibition might be a safe and effective strategy to counteract age-related kidney pathology (Di Francesco et al., 2018) in multiple cellular processes such as those implicated in nutrient and energy-sensing pathways (e.g., AKT, TOR, and S6K) also in that case, with an intermittent administration (Hofmann et al., 2015).

#### Metformin

Metformin is the most commonly prescribed medication for type 2 diabetes in the world. This drug has been associated with reduction in the risk of cardiovascular events and can be translated in the treatment of cancer for its ability of inhibiting metabolism or reducing stemness in cancer cells (Deschênes-Simard et al., 2019). Recently, metformin was evaluated for its renoprotective capacities, thus since 2016 the Food and Drug Administration (FDA) allowed the utilization in patients with CKD even without the diabetic kidney disease (Ravindran et al., 2017). More importantly, these drugs have beneficial effects on aging and life span extension (Barzilai et al., 2016). The proposed mechanism of SASP inhibition has been identified in NF-κB signaling inhibition, and Nrf2 modulation (Moiseeva et al., 2013; Fang et al., 2018; Kanigur Sultuybek et al., 2019).

Furthermore, metformin reduces ROS formation, γ-H2AX foci, and ATM, reducing the formation of senescent cells (Park and Shin, 2017). Because of such compelling evidence, a study was launched in consultation with FDA (Barzilai et al., 2016) aiming at the approval of an additional therapeutic indication for metformin. The study is called TAME (Targeting Aging with MEtformin) and is an inspiring example of how drug repurposing may contribute to the development of new therapeutic approaches (Fang et al., 2018; Kulkarni et al., 2020).

## Future Perspective in Senotherapy: Application in Transplantation Field

**FIGURE 2:**
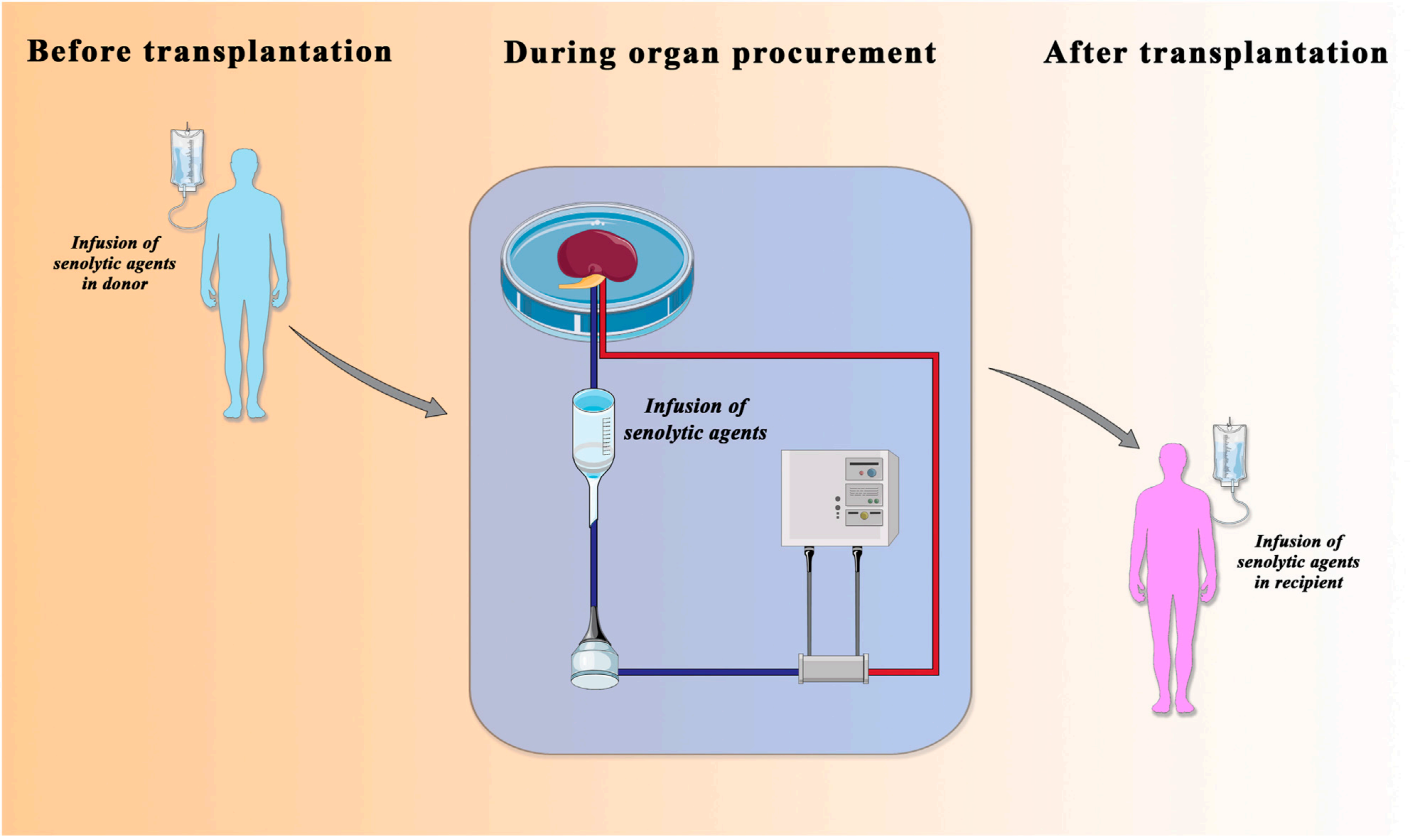
Senolytic agents in transplantation field. The administration of senolytic agents in donor **(left)**, in normothermic-perfusion device **(middle)**, and in recipient **(right)** is considered a therapeutic approach to improve the quality of older donor organs that have traditionally been considered unusable for transplantation. Adapted from Lau A, Kennedy BK, Kirkland JL, Tullius SG. Mixing old and young: enhancing rejuvenation and accelerating aging. J Clin Invest. 2019 Jan 2; 129 (1), 4–11.

Renal transplantation is a lifesaving treatment for patients with ESRD (Carminatti et al., 2019). However, although renal transplantation can dramatically impact the quality of life and save expensive dialysis costs there are still unsolved problems. First, there are insufficient numbers of donor kidneys available and more than 20 people die every day while waiting for a transplant organ. The ongoing donor kidney shortage is a prominent issue for transplant centers worldwide and forces transplant professionals to accept “marginal” organs from cardiac death or older (age ≥60) donors, namely, the DCD (donation after circulatory death) and ECD (expanded criteria donors) donors. However, the use of these kidneys correlates with a poorer survival, incidence of rejection, and delay graft function (DGF) (Gavriilidis and Inston, 2020). Furthermore, due to increasing average age of the donors, the number of these discarded and marginal organs is sharply increasing. Second, regardless of donor age, the transplant surgery itself, with the unavoidable risk of IRI, can massively affect organ quality. The pathophysiology of renal IRI is complex and involves the interplay between extensive release of pro-inflammatory cytokines by polymorphonuclear leukocytes, oxidative stress with lipid peroxidation, imbalance of coagulation and fibrinolysis, the endothelial dysfunction with increased adhesion molecules, and deranged NO production (Franzin et al., 2020; Salvadori et al., 2015; Castellano et al., 2016). If these pro-inflammatory responses are not balanced, they can lead to premature renal aging. Recently, machine perfusion technology was identified as a strategy to improve outcomes from these grafts, in particular through the pharmacologic targeting of IRI-associated premature aging. The use of machine perfusion demonstrated superior outcomes in early allograft dysfunction compared to Static Cold Storage. Recent studies have shown that *ex vivo* normothermic machine perfusion (NMP) led to lower rates of DGF, improved renal metabolism, and reduced renal IRI. NMP appears to be superior as a preservation method, thus potentially increasing the donor pool by improving the outcome of transplantation of grafts from ECD as well as from DCD. Besides allowing the kidney to regain its function, NMP can be used to keep the kidney in a controlled state allowing close observation and viability assessment enabling successful transplantation of declined kidneys. From a renal aging perspective, machine perfusion provides the window of opportunity to add therapies to a functioning organ extracorporeally (Krzywonos-Zawadzka et al., 2019; Hosgood et al., 2020) (Figure 2). Together with gene-manipulating therapies, nanoparticles, anti-inflammatory agents, anti-thrombolytic agents, and monoclonal antibodies directed against complement activation (Jager et al., 2017), senotherapeutic agents could represent an important strategy of intervention.

Moreover, the production of artificial vesicles, which can transfer therapeutic miRNAs to target tissue and cells, could be a new therapeutic strategy to facilitate tissue regeneration and counteract aging-associated diseases.

In particular, the natural EV derived from mesenchymal stem cells have been proposed as a new strategy to improve graft survival, thanks to the modulation of tolerance toward the graft, and to their anti-fibrotic, anti-inflammatory, and antiaging effects (Grange et al., 2020). Moreover, MSC-EVs may reduce ischemia reperfusion injury, improving recovery from acute damage. A recent application that combined the MSC-derived EV with the machine perfusion in the context of kidney transplantation has been provided by Gregorini et al. (2017). The supplementation of MSC-EV in perfusion solution during renal treatment preserved kidney function as assessed by histological and genetic analyses on EV-treated kidneys.

In conclusion, senolytics agents may alleviate renal senescence‐associated complications (i.e., diabetes nephropathy and vascular dysfunction leading to CKD); however, they still hold a controversial position in the regulation of healthy renal aging.

## Abbreviations

AKI, acute kidney injury; ESRD, end-stage renal disease; CKD, chronic kidney disease; IRI, ischemia reperfusion injury; SASP, senescence-associated secretory phenotype.
